# 

**DOI:** 10.1038/sj.bjc.6600377

**Published:** 2002-01-02


© 2002 Cancer Research UK

a dominant band at 80kD and a weaker band at 120kD were detected. CD44v6 protein expression was not altered in activated cells. Expression profiles of other adhesion moecules are under investigation. Thus natural ligands of CD44 ie hyaluronate, have a marked effect on expression of other families of adhesion molecules whereas activation by specific antibody, while causing upregulation of MMP's, does not have as profound an effect on the adhesion molecules investigated to date.

**P134**

**ERK5 EXPRESSION IN HUMAN PROSTATE CANCER**

B.L.Jenkins*, P.B.Mehta, C.N.Robson, D.E.Neal and H.Y.Leung.
Department of Surgery, Medical School, University of Newcastle-Upon-Tyne, NE2 4HH

**Introduction**

ERK5 is required for EGF-induced cellular proliferation and progression through the cell cycle[1]. We have recently shown that MEK5, the specific activator of ERK5, is over-expressed in prostate cancer and is associated with metastatic disease and poor survival. ERK5 has three splice variants, namely a, b, and c, encoding for different amino termini[2]. The full length ERK5a is catalytically active, whereas the b and c forms are dominant negative kinases. We studied the expression of ERK5 isoforms, to further characterise the function of the MEK5/ERK5 pathway in human prostate cancer.

**Materials and Methods**

Primers flanking the region in which ERK5 varies in the three splice isoforms were designed. RNA was extracted from a panel of human prostate, bladder, breast and colon cancer cell lines. It was then subjected to reverse transcription PCR analysis. Snap frozen resected prostatic (7 BPH, 9 Cancer) tissue were also studied. Western blotting was performed on four of the cell lines to confirm ERK5 expression.

**Results**

The amplicons seen were related to the presence and size of the intron insert at the region of interest; a-115bp, b-175bp, c-251bp. In J82 bladder cancer cells, all three ERK5 isoforms were detected with the 'a' and 'c' forms predominant. In the other cell lines examined, only the ERK5 'a' variant was detected. Uniform ERK5'a' expression was also seen in the benign and malignant prostate tissue throughout all the samples. Western blotting of the cell lines exhibited the same expression pattern as was seen in the PCR results.

**Discussion and conclusion**

In murine tissue all three ERK5 isoforms are expressed in testis, brain, kidney, lung and heart. The expression of the 'a' and 'c' forms are much stronger than the 'b' form. We have shown that ERK5 'a' is expressed in human prostatic tissue, and the dominant negative proteins and were not detected. The 'b' and 'c' isoforms may be suppressed in active tissue allowing excessive growth seen in both BPH and prostate cancer. It is possible that manipulation of the expression of these splice variants could be performed to inhibit excessive growth.

[1] Kato Y, Tapping RI, Huang S et al. 1998 *Nature* 395: 713 [2] Yan C, Luo H, Lee JD et al. 2001 *J Biol Chem* 276: p10870

**P135**

**MOLECULAR AND MUTATION ANALYSIS OF HEREDITARY MULTIPLE EXOSTOSES**

Wing-Sum Hui[1], Kathryn S.E. Cheah[1], Jing-Dong Huang[1], Kenneth M.C. Cheung[2], Liu Yang[3], Z.J. Luo[3], Ravi Savarirayan[4] and Danny Chan[1]
Department of Biochemistry[1] and Department of Orthopaedic Surgery[2], The University of Hong Kong, Hong Kong, China, Department of Orthopaedic surgery[3], The Fourth Military Medical University, X'ian, China, and Genetic Health Services Victoria[4], Melbourne, Australia

Hereditary multiple exostoses (HME/EXT; MIM133700) is an autosomal dominant disorder characterized by the presence of multiple benign cartilage-capped bone tumors. It has been ascribed to mutations in two tumor suppressor-related genes, *EXT1* and *EXT2*, encoding glycosyltransferases required for heparan sulfate proteoglycan (HSPG) synthesis. The molecular basis for tumor formation is not known but a number of hypotheses have been proposed that center on the alteration of HSPG and deregulated endochondral bone formation. These included altered Indian hedgehog (Ihh) signaling or fibroblast growth factor (FGF) function because diffusion of Ihh is thought to be regulated by HSPG, and FGF function requires binding to haparin. Ihh and FGF are important molecules that regulate chondrocyte differentiation and proliferation in endochondral bone formation.

To gain insight into the molecular basis for HME/EXT, we analyzed seven patients; two from Australia, four from Hong Kong and one from Mainland China for mutations in *EXT1* or *EXT2* genes. A novel mutation of a single nucleotide deletion (386-388 delA) in exon 1, was identified in *EXT1* in an Australian patient. This resulted in shift of the codon reading frame from serine[130] with a predicted termination at position 134. In the other Australian patient, a previously reported mutation, G679A (D227N), in exon 2 of *EXT2* was identified. A 1173+1 G→T FS R360 mutation was identified in the Mainland patient in the *EXT2* gene. This mutation represents a nucleotide substitution of guanine to thymine at position 1173 at the +1 position of intron 7. This mutation alters the 5' splice site and the most likely consequence is the splicing out of exon 7, resulting in a shift of the codon reading frame from arginine at position 360 (R360) of the amino acid sequence. No mutation in either *EXT1* or *EXT2* was detected for the four patients from Hong Kong.

Analysis of tumor samples obtained from Hong Kong and Mainland patients are in progress to study changes in cartilage differentiation using immunostaining and *in situ* hybridizaion. Markers for chondrocyte differentiation and proliferation are being assessed together the localization of haparan sulfate and Ihh. Preliminary data showed chondrocyte differentiation and cellular organization differs to that of a normal growth plate. This study will provide insight into possible mechanisms, and a direct comparison of cellular changes between bone tumors with or without mutations in the *EXT1* or *EXT2* genes.

**P136**

**IDENTIFICATION OF NOVEL CANDIDATE GENES WITHIN THE 250KB TYLOSIS WITH OESOPHAGEAL CANCER (TOC) MINIMAL REGION ON CHROMOSOME 17q25**

Fiona McRonald*[1], Janet Risk[1], Tony Ellis[3], Lynn Rowbottom[1], Joanne Langan[1], John Field[1,2]
1 Molecular Genetics and Oncology Group, Dept. of Clinical Dental Sciences, University of Liverpool, Liverpool L69 3GN
2 Roy Castle International Centre for Lung Cancer Research, University of Liverpool
3 Dept of Gastroenterology, Royal Liverpool University Hospital, Prescot Street, Liverpool

The association of autosomal dominant palmoplantar keratoderma (tylosis) and oesophageal cancer has been recognised in three families. Linkage analysis has mapped the TOC gene to a 250kb region on chromosome 17q25. The tissue-specific expression profile of four known genes within this region was investigated by RT-PCR, and all of these genes were shown to be active in the tissues of interest, namely skin, oesophagus, and squamous cell oesophageal cancer.

The entire 250kb region has been examined using the online bioinformatics package NIX (Nucleotide Identification X) available through the UK Human Genome Mapping Project Resource Centre. Expression of regions of interest (predicted exons and ESTs) identified by NIX was investigated by RT-PCR, and evidence for seven novel genes within the TOC region was obtained. All of these genes appear to be expressed in the tissues of interest. One of these genes has since appeared in Genbank and has been screened for mutations in the TOC families, and mutation analysis is underway on a further single-exon gene that is registered in Genbank.

Further characterisation of the remaining RT-PCR gene fragments, by IMAGE clone sequencing and cDNA library screening, will aid in the identification of the TOC gene by providing full-length candidate genes for mutation analysis. The TOC minimal region has been shown to be implicated in sporadic as well as familial oesophageal cancer. The prognosis for patients with squamous cell oesophageal cancer is generally poor, often due to late presentation of the tumour. Identification of the TOC gene could potentially have applications in novel diagnostic and therapeutic strategies for oesophageal cancer.

© 2002 Cancer Research UK

**P137**

**VARIATIONS IN THE EXPRESSION OF P53 AND DOWNSTREAM TARGET GENES P21 AND MDM2 IN BREAST CANCER TREATMENT**

D Ziyaie*[1], TR Hupp[2], NM Kernohan[2], A Ingram[1], AM Thompson[1]. [1]Dept. of Surgery & Molecular Oncology, [2]Dept. of Molecular & Cellular Pathology, Ninewells Hospital & Medical School, Dundee DD1 9SY.

**Background:** In the treatment of breast cancer, clinical studies have shown that tumours which are clinically and histologically similar vary in their response to chemotherapeutic drugs. The mechanism of action of cytotoxic drugs is complex but in general mediated through induction of DNA damage and hence activation of p53 and it's downstream pathway. Two key gene products of p53, p21WAF1 and MDM2, are known to be transcriptionally induced by p53. p21 mediates the tumour suppressing effects of p53 and the MDM2 protein controls the biological activity of p53 and targets it for destruction.

**Aims:** To examine the relationship between p53 and its key effector genes p21 and MDM2 in primary breast cancers treated with neo-adjuvant chemotherapy.

**Methods:** For 26 patients with breast cancer, p53, p21 and MDM2 protein expression in response to chemotherapy treatment was determined by western blot analysis using highly sensitive human specific monoclonal antibodies. The results were compared with pathological data available on the patients following surgery and the clinical data available during a minimum three years follow up with full approval of the Local Ethical Committee.

**Results:** Evidence of activity in the p53-MDM2 pathway was observed in tumours that showed a more favourable response to treatment and overall prognosis. No distinct relationship was seen between tumour behaviour/final outcome and p53-p21 axis as presence or absence of activity in this pathway had no clear link to the clinical/pathological variables.

**Conclusion:** The data suggest the p53-MDM2 pathway is activated in breast cancers with more favourable prognosis. More detailed analysis may provide the framework for novel anti-cancer drugs that target the specific proteins involved.

**P138**

**COMBINATION OF MICRODISSECTION AND MICROARRAY ANALYSIS TO STUDY GENE EXPRESSION CHANGES IN BREAST CANCER INVASION**

G. Zhu*[1], T. Crnogorac-Jurcevic[2] and IR. Hart[1]. [1]Richard Dimbleby Department of Cancer Research/ ICRF Labs, St Thomas' Hospital, London SE1 7EH, [2]ICRF Molecular Oncology Unit, Hammersmith Hospital, London W12 0HS

Gene expression changes may play an important role in cancer invasion. To evaluate this possibility, the PALM micro-laser dissection and capture system was employed to isolate pure populations of tumour cells from two breast cancer types; invasive cancer and ductal carcinoma in situ (DCIS). We dissected the central and peripheral areas of these tumour lesions separately and pooled cancer cells from 10 sections, which were obtained from 5 different patients. By using the TRZoL reagent, total RNA was extracted from microdissected tissue. The Atlas SMART™ Probe Amplification Kit was used to synthesise and amplify cDNA. Probes from the periphery and from the centre were hybridised to duplicates of Atlas Human Cancer 1.2 Arrays which contain 1,176 known genes. After comparing images by AtlasImage™ 2.0, we have found that 22 genes changed their expression levels in the periphery of tumour lesions relative to the central regions of these tumours. 15 genes are up-regulated, and 7 genes are down-regulated ( arbitrary threshold of 1.5-fold or greater ). Verification that this procedure was identifying genes likely to be of importance in tumour invasion was provided by the observed up-regulation of the mesenchymal marker vimentin at the periphery of the tumours. Equally there was up-regulation of Rho C, which already has been shown to have higher expression levels in metastases relative to the primary tumour. TSG 101 and EF-1 α were shown , by microarray analysis, to be expressed differentially between centre and periphery. Using immunohistochemistry, we have been able to confirm these findings at the protein level.

The precision of the PALM microdissection technique allows the resolution of cDNA microarray gene expression analysis to be increased from the level of tissue analysis to analysis of one or more of its component cell populations. Using this method we have identified changes in gene expression associated with varying micro-anatomical locations within the developing tumour mass.

**P139**

**THE CHERNOBYL THYROID TUMOUR BANK – AN INTEGRATED APPROACH TO RESEARCH ON CANCER**

GA Thomas, South West Wales Cancer Institute, Singleton Hospital, Sketty, Swansea SA2 8QA, on behalf of the Scientific Project Panel of the NISCTB

The only unequivocal effect of the Chernobyl accident on human health shown to date is the very large rise in incidence of thyroid carcinoma in children, in particular papillary carcinoma. Although the post Chernobyl thyroid tumours are associated with a particular aetiology, they may also provide general insights into the pathogenesis and molecular biology of other cancers. However, up to now, one of the major problems for research has been access to a large number of well documented samples.

The NISCTB (Newly Independent States Chernobyl Tumour Bank; www.srl.cam.ac.uk/nisctb) is a unique venture. It is the first international cooperation that seeks to establish and make available to the world-wide research community a collection of biological samples from tumours and normal tissues from patients for whom the aetiology of their disease is known – exposure to radioiodine in childhood. Full ethical permission (overseen by the NCI of the USA) was obtained for all samples held. In order to maximise information from small samples, the NISCTB supplies extracted nucleic acids rather than pieces of tissue for individual researchers. Quality control by multiplex RT-PCR or PCR is carried out on each tissue extract. A full description of the specimen; its location within the thyroid; whether other tumours are present in the gland and the relative locations of material from which RNA and DNA is extracted after frozen section confirmation, together with an internationally agreed diagnosis is recorded on the project database. The project has been collecting material since 1st October 1998; frozen material is available on 935 of 1161 cases and aliquots of RNA and DNA are currently available from 421 paired samples of tumour and normal tissue; DNA from whole blood and aliquots of serum is also available on over a third of these cases in the near future. In addition to information relating to the biological material, documentation and tracking of the specimens themselves, basic clinical information is also recorded on each patient in the project database.

Material from the project is currently being used amongst other things to study aspects of cell cycle regulation, expression of tyrosine kinase genes using cDNA array and identification of ret gene rearrangement. In the future this will permit results from molecular biological studies on different oncogenes from separate studies to be correlated to investigate possible gene interactions. This project could become a paradigm for investigations on the mechanisms of human cancer, and provide a basis that fosters international collaboration in cancer research. The investigations carried out on the material from the NISCTB will undoubtedly provide information not only on mechanisms of thyroid carcinogenesis, but also information on fundamental aspects of human cancer.

**P140**

**MODULATION OF THE CALPAIN-CALPASTATIN PROTEOLYTIC SYSTEM AND CALPAIN-MEDIATED DISASSEMBLY OF CELL-CELL AND CELL-MATRIX ADHESIONS IN METASTATIC EPITHELIAL CELLS**

N.O. Carragher[¶], M.A. Westhoff[¶] and M.C. Frame[¶‡]

[¶]The Beatson Institute for Cancer Research, Cancer Research Campaign Beatson Laboratories, Glasgow G61 1BD. [‡]Institute of Biological and Life Sciences, University of Glasgow, Glasgow G12 8QQ.

Tumour metastases is dependent on the disruption of both cell-cell and cell-matrix adhesions. Intercellular and extracellular matrix adhesions are primarily governed by cadherin-dependent adherens junctions and integrin-linked focal adhesion attachment sites respectively. Disruption of these cytoskeletal linkages is associated with enhanced cell motility and tissue matrix invasion. Recent studies suggest calpain-mediated proteolytic degradation of key components of the cadherin and focal adhesion complexes

 © 2002 Cancer Research UK

may represent a mechanism for disassembly of these complexes and subsequent loss of cell-cell and cell-matrix adhesions. The calpains represent a highly conserved family of non-lysosomal calcium dependent cysteine proteases. *In vivo* calpain activity is tightly regulated by its highly specific endogenous inhibitor, calpastatin. We have recently demonstrated that calpain mediated proteolytic cleavage of the focal adhesion kinase (FAK) and subsequent disassembly of the focal adhesion complex accompanies v-Src induced morphological transformation of fibroblasts contributing to increased cell motility (Carragher *et al*., 2001. *J.Biol.Chem.* **276**:4270). Increased calpain activity in v-Src transformed fibroblasts also promotes cell cycle progression and anchorage-independent growth (Carragher *et al*., 2002. *Mol. Cell.Biol.* **22**:257). To further establish the role of calpain in tumour cell metastasis we have examined the role that calpain plays in regulating the turnover of the adhesive complexes of metastatic epithelial cells. We have demonstrated that the protein levels of calpain are often upregulated in parallel with decreased levels of calpastatin in metastatic epithelial cell lines relative to non-metastatic controls. Furthermore, using pharmacological inhibitors we have demonstrated that inhibition of calpain activity stabilizes integrin-linked focal adhesion complexes of both non-metastatic and metastatic epithelial cells. Inhibiting calpain activity also suppresses the disassembly of cadherin-dependent cell-cell adhesions in response to culture of epithelial cells in low calcium conditions. These studies indicate that modulation of calpain activity may contribute to the turnover of integrin-linked focal adhesions and cadherin-dependent adherens junctions in metastatic epithelial cells.

**P141**

**EXCISION REPAIR CROSS-COMPLEMENTING GENE 1 (ERCC1) PROTEIN EXPRESSION IN COLORECTAL CANCER**

SD Richman[1]*, JW Adlard[1], MT Seymour[1], P Quirke[2]. [1]Cancer Research UK. [2]Pathology, University of Leeds.

**Introduction:** The ERCC1 gene is located on chromosome 19 and encodes one of the major enzymes involved in nucleotide excision repair (NER)[1]. NER is the mechanism of repair of DNA adducts caused by ultraviolet radiation and platinum-based chemotherapies. High levels of tumour ERCC1 mRNA expression have been linked with resistance to cisplatin in gastric cancer[2] and oxaliplatin in colorectal cancer[3]. Determining the levels of mRNA expression is a complicated technique not available to most histology departments. We have developed a protocol for determining ERCC1 protein expression using immunohistochemistry (IHC) which may be a more practical alternative.

**Methods:** Archival formalin-fixed, paraffin-embedded specimens were retrieved from 224 consenting patients in the MRC FOCUS clinical trial of chemotherapy for advanced colorectal cancer. Tissue microarrays (TMAs) were produced containing 0.6mm cores of tumour and where available, corresponding normal colonic mucosa. 0.5micron sections of TMAs were cut for IHC. Very small biopsies were examined as individual whole sections. A reproducible protocol was developed for IHC using a mouse monoclonal antibody to the human ERCC1 protein (Lab Vision-Neomarkers, Fremont, California). Staining was scored by two independent assessors as positive or negative, and the pattern of expression was recorded.

**Results:** 224 colorectal cancers and 138 corresponding normal colonic mucosa specimens were assessed. 28% of normal mucosa and 33% of cancers were positive for ERCC1 protein expression. 76% of normal tissues stained with a nuclear pattern, 21% with a cytoplasmic pattern and 3% with both nuclear and cytoplasmic staining. For tumour tissue, the rates were 36%, 59% and 5% respectively. Staining of whole sections, including both normal and malignant tissue, identified cases where normal tissue showed nuclear staining and adjacent tumour tissue showed cytoplasmic staining. Different patterns of cytoplasmic staining were observed in cancers, varying from a diffuse pattern to very discrete granules.

**Conclusions:** Immunohistochemical staining for ERCC1 using this technique gives positive staining for a third of colorectal cancers. We noted a tendency for positive normal colonic tissue to show predominantly nuclear staining and for cancers to show cytoplasmic staining. ERCC1 protein is produced in the cytoplasm and transported to the nucleus. Some colorectal cancers may have dysregulation of ERCC1 protein production or transport. We are continuing to assess the role of ERCC1 protein expression as a predictor for response to oxaliplatin chemotherapy.

**References**:

[1]McHugh PJ *et al* (2001). *Lancet Oncol* 2:483.
[2]Metzger R *et al* (1998). *J Clin Oncol* 16:309.
[3]Shirota Y *et al* (2001). *J Clin Oncol* 23:4298.

**P142**

**THE MRC CR08 (FOCUS) TRIAL MOLECULAR PATHOLOGY PROJECT. IDENTIFYING PREDICTIVE FACTORS FOR CHEMOTHERAPY RESPONSE.**

JW Adlard[1]*, SD Richman[1], R Stephens[2], A Meade[2], S Hassan[2], P Quirke[3], MT Seymour.[1] For the FOCUS Trial Investigators. [1]Cancer Research UK. [2]Medical Research Council. [3]Pathology, University of Leeds.

**Introduction:** After over 40 years of 5-Fluorouracil (5FU)-based chemotherapy for colorectal cancer, new agents are now available, including irinotecan and oxaliplatin. However, we are still unable to reliably identify factors that will allow selection of a chemotherapy regimen for an individual patient that will definitely be of benefit. Reported data on molecular predictive factors are so far based mainly on small numbers of patients, outside the setting of randomised clinical trials.[1] Even with the most studied marker for 5FU response, thymidylate synthase (TS), there are conflicting accounts of its value. The MRC CR08 (FOCUS) Trial is investigating the role of irinotecan and oxaliplatin and their sequencing, in addition to 5FU-based chemotherapy for 2100 patients with advanced colorectal cancer[2] and is therefore an excellent opportunity to study predictive molecular variables in a controlled clinical setting. FOCUS is still recruiting and includes a request to retrieve stored pathological material for bowel cancer research.

**Methods:** Over 95% of patients entering FOCUS have consented to this study. Archival primary tumour and when available, metastasis biopsy specimens are retrieved in anonymised form. Tissue microarrays (TMAs) are produced with multiple tumour and normal tissue cores from each patient and these are used for immunohistochemistry (IHC) to assess protein expression. Potential markers being tested by IHC include: for 5FU; TS, thymidine phosphorylase (TP), dihydropyrimidine dehydrogenase (DPD), for irinotecan; topoisomerase 1 and for oxaliplatin; excision repair cross-complementing gene 1 (ERCC1). We are also investigating RNA expression of the same markers using real-time reverse transcriptase (RT-) PCR. Candidate DNA markers for allelic imbalance, microsatellite instability and gene polymorphisms are also being examined. The role of cDNA microarrays in identifying novel predictive factors is being investigated.

**Results:** To date 300 sets of tumour blocks have been retrieved for study. Techniques being used and a report of the progress of the project will be presented at the meeting. An initial assessment of TS expression using IHC in 229 cases, all receiving 5FU (67% alone; 33% in combination with irinotecan or oxaliplatin), has shown that 28% of tumours are negative (Grade 0/1) and 72% are positive (Grade 2/3). In this preliminary group, TS staining does not correlate with the clinical response status after the first 3 months of chemotherapy (P=0.37).

**Conclusions:** This study is demonstrating that large-scale retrieval and processing of surplus pathological material in association with randomised clinical trials is feasible, and gains almost universal patient consent. The large number of patients in FOCUS will allow chemopredictive association to be examined using a range of candidate markers, with greater statistical power than previously possible.

**References:**

[1]Adlard J.W. *et al* (2002). *Lancet Oncol* 3:75.
[2]MRC CR08 Clinical Protocol (Version 5) June 2001.

**P143**

**IMMUNOHISTOCHEMICAL PROFILING OF PATHWAY ACTIVATION IN TUMORS USING ACTIVATION-SPECIFIC ANTIBODIES.**

Katie Crosby, Michael Melnick and Bradley L. Smith, Cell Signaling Technology, Beverly, MA

We have used phospho-specific antibodies to characterize the activation status of a broad range of therapeutically relevant signal transduction pathways in human breast cancer tissues by immunohistochemical

© 2002 Cancer Research UK

assessment of tissue arrays. We validated the phospho-specific antibody immunohistochemical (IHC) results with methods including Western blot analysis, antigen absorption and staining of embedded cells. Akt kinase pathway activation was demonstrated by IHC with prostate tumor-derived LNCaP cultured cells, which lack PTEN phosphatase. IHC analysis of LNCaP cells also demonstrated the induction of apoptosis following inhibition of the Akt pathway. Multi-pathway IHC analysis of human breast tumor tissue arrays showed significant correlations among activation states of diverse signaling proteins and implicated multiple pathways underlying the neoplastic phenotype. Cluster analysis of protein activation suggests that key signaling pathways cluster with EGFR overexpression. In contrast, HER2 overexpression failed to cluster with any of the pathways tested. The activation state of specific signaling proteins was also found to correlate with pathological indices and patient prognosis. This is illustrated by the significant correlation between the activation states of Erk/MAPK and estrogen receptor, and breast cancer pathological indices of tumor grade and lymph node status. The ability to profile the activation status of a specific therapeutic target, as well as those of multiple other signaling proteins will be an important complement to the new generation of targeted therapeutics and will enable effective and precise diagnosis of the molecular basis of disease.

**P144**

**TARGETED INTERRUPTION OF THE CDK4-CYCLIN D1 COMPLEX**

Margaret Crawford[1]* Barbara Saxty[2], Catherine Kettlebourough[2], Paul Ko Ferrigno[1]
[1]Hutchison MRC Building, Addenbrooke's Site, Hills Rd, Cambridge, CB2 2XZ
[2]MRC Technology Assay Development Group, 1-3 Burtonhole Lane, Mill Hill, London, NW7 1AD

Cyclin dependent protein kinases (CDKs) control cell cycle progression and thus provide a good target for the development of inhibitory molecules for cancer therapy. To date inhibitors have been directed against the kinase domain of each CDK. We aim to develop inhibitors that target the binding of CDK4 to cyclin D, an interaction which is essential for CDK4's activity. In addition to inhibiting CDK4's ability to support cell cycle progression, our inhibitors are also likely to facilitate CDK4-independent activities of cyclin D, which are in general themselves thought to be anti-proliferative. This two pronged approach should have broad applicability in the clinic.
To screen for small molecules that would inhibit the formation of the cyclin D/CDK4 complex, we are using a peptide aptamer that defines the cyclin D binding site. Peptide aptamers are themselves small molecules, but ones that can be readily screened in a yeast two-hybrid format for those that bind to a protein of interest. Because of their biological (peptidic) nature, peptide aptamers are particularly useful in disrupting protein-protein interactions in vivo. A random peptide aptamer library consisting of peptides (20 amino acids long) was screened against cyclin D (see accompanying abstract). One of the biologically active peptide aptamers was then used as the basis for the following screen.
We are establishing a small molecule screen in a cell free system using bioluminescence resonance energy transfer (BRET) technology. We intend to screen a small diverse compound library (10, 000 compounds) to identify inhibitors of the cyclin D/CDK4 complex. Results will be presented.

**P145**

**THE ADDITION OF ALL-TRANS RETINOIC ACID (ATRA) COUNTERACTS THE INCREASE IN BCL-2 LEVELS INDUCED BY CYTOTOXIC AGENTS**

K.Scatchard, K.Makrogianelli, WM. Liu, L.Goff, AZR.Rohatiner and S.Joel.
Cancer Research UK Dept Medical Oncology, St Bartholomew's Hospital, London.

**INTRODUCTION**: Over-expression of BCL-2 is common in AML and is associated with increased resistance to cytotoxic drugs. ATRA has been shown to down regulate BCL-2 levels and may thus improve chemosensitivity. We investigated the effects of combining ATRA with existing anti-leukaemic agents on BCL-2 expression and cytotoxic response in a panel of human leukaemic cell lines.
**METHODS**: CEM, HL60, K562 and ML-1 cells were exposed for 4 days to $IC_{25}$ concentrations of cytarabine (C), idarubicin (I) or etoposide (E) alone and in combination with 10 µM ATRA (A, previously shown to be optimal for BCL-2 downregulation). Cell viability and % apoptosis were assessed daily. BCL-2 status was assessed by flow cytometry, immunoblotting and 'real-time' PCR.
**RESULTS**: Exposure to low concentrations of A, I or E alone typically raised BCL-2 levels (in CEM cells on day 4, with 10 nM C or 3 nM I: +20 ± 9% and +22 ± 4%, respectively; p<0.025). Conversely, A alone resulted in decreased BCL-2 by day 1, which was maintained through to day 4 (-20 ± 6% on day 1 and -14 ± 8% on day 4; p<0.02). The combination of ATRA with cytotoxic agents typically resulted in BCL-2 levels that were lower than those seen with cytotoxic agents alone (at day 4 in HL60 cells: +1 ± 15% with C alone –52 ± 6% with C+A; p=0.038). The changes in BCL-2 protein correlated with changes in *bcl-2* mRNA and were similar for CEM and ML-1 cells. % viability for each cytotoxic agent ± A are shown in the table. Flow cytometric analysis indicated cell death to be by apoptosis.

| TREATMENT | %VIABILITY DAY 4 mean ±SD | | | |
|---|---|---|---|---|
| | CEM | HL60 | K562 | ML1 |
| Control | 98 ± 1 | 96 ± 0 | 98 ± 2 | 95 ± 1 |
| A | 90 ± 4 | 75 ± 4 | 94 ± 4 | 82 ± 6 |
| C | 87± 6 | 75 ± 7 | 75 ± 4 | 91 ± 5 |
| C+A | 67 ± 3 | 45 ± 12 | 64 ± 12 | 74 ± 7 |
| I | 41 ± 4 | 35 ± 15 | 60 ± 10 | 43 ± 18 |
| I+A | 21 ± 6 | 15 ± 13 | 60 ± 6 | 46 ± 22 |
| E | 43 ± 4 | 46 ± 6 | 66 ± 1 | 85 ± 2 |
| E+A | 36 ± 10 | 34 ± 17 | 72 ± 8 | 58 ± 11 |

**CONCLUSIONS**: These data confirm the increased cell kill reported when ATRA is used with cytotoxic agents, but this increased cell kill is no greater than additive. Whilst ATRA prevents the rise in BCL-2 seen with chemotherapy alone this is insufficient to cause a synergistic increase in cell kill. As ATRA effects the expression of many other genes, agents that target BCL-2 more specifically, such as Antimycin $A_3$ may help to determine whether targeting BCL-2 sensitises cells to conventional agents.

**P146**

**THE DETERMINATION OF HISTONE DEACETYLASE ACTIVITY IN INTACT CELLS USING A NON-ISOTOPIC ASSAY**

Simon Joel[1], Catherine Mikol[1], Charles Marson[2], Jackie Perry[1], Alphonso Rioja[2], Simon Clark[1], Bryan Young[3]. Department of Medical Oncology[1], St Bartholomew's Hospital, London, Dept of Chemistry[2], University College, London, Cancer Research UK[3] Molecular Oncology Lab, St Bartholomew's Hospital, London

Inhibition of histone deacetylases (HDAC's) may have therapeutic potential in patients with malignant disease. A rapid *in vitro* HDAC activity assay, based on the deacetylation of an [illegible]acetyllated lysine (MAL), has recently been described (Hoffmann et al, Nuc Acids Res 1999; 27: 2057-8), with substrate and product (ML) concentrations determined by specific HPLC analysis with fluorescence detection. We have further developed this assay for use in intact cells with a single time-point reaction. For isolated rat liver, enzyme, substrate (5 µg/mL MAL) and inhibitor at 6 conc$^{\underline{n}}$ were incubated at 37°C for 60 minutes, after which the reaction was stopped with acetonitrile, and MAL and the deacetylated product ML determined in the supernatant. For intact cells, $1x10^6$ CEM cells in 1 mL medium were exposed to inhibitors at 6 conc$^{\underline{n}}$ for 60 minutes, after which MAL (20 µg/mL) was added for a further 30 minutes, all at 37°C. Cells were then rapidly washed at 4°C, lysed by sonication, the reaction stopped and MAL and ML determined in the supernatant. The $IC_{50}$ values for % viability were determined over a 3-day continuous exposure in CEM cells. Results are presented in the table for phenylbutyrate (NaPB), phenylbutyrate hydroxamic acid (NaPBHA) and Trichostatin (TSA)

© 2002 Cancer Research UK

| | HDAC inhibitory activity $IC_{50}$ | | % Viability $IC_{50}$ |
|---|---|---|---|
| | Rat liver | Intact CEM cells | CEM cells |
| NaPB | 405 µM | 9.5 mM | 8.4 mM |
| NaPBHA | 3.1 µM | 269 µM | 244 µM |
| TSA | 19 nm | 23 nM | 119 nM |

The relative HDAC inhibitory activities of NaPB and NaPBHA are in line with the proposed structure of the enzyme active site. Although more time intensive, this modification of the HDAC activity assay for use in intact cells may provide a better indication of the potential biological activity of selected compounds.

**P147**

**A CELL-BASED ELISA IS A USEFUL PHARMACODYNAMIC AND SCREENING TOOL TO EVALUATE CHANGES IN CELLULAR ACETYLATION**

Lindsay Stimson*, Yvette Newbatt, Anthea Hardcastle, Kathy Boxall, Paul Workman, G Wynne Aherne. Cancer Research UK Centre for Cancer Therapeutics, Institute of Cancer Research, 15 Cotswold Road, Sutton, Surrey, SM2 5NG

Acetylation of specific lysine residues in histone tails is an essential element (with phosphorylation and methylation) of an intricate histone code that regulates gene transcription in a temporal and context dependent manner. Acetylation is regulated by two classes of enzyme, histone acetyltransferases (HATs) and histone deacetylases (HDACs). Several HDAC inhibitors have been described and some are presently in pre-clinical and clinical development. There is also evidence that deregulated HAT enzymes play a role in the progression of some cancers. No drug-like inhibitors of HATs have yet been described. High-throughput screening is being used in the Centre to screen a ~60,000 compound collection for compounds that inhibit HATs. Such compounds would be useful chemical probes to investigate the effects of HAT inhibition on cellular behaviour and could form the basis of a new type of chemotherapy. It is important that a mechanism-based assay with relatively high throughput is available to compare compounds identified by screening. A cell-based ELISA (cytoblot) has been used as an alternative to Western blotting for determining the effects of HDAC and HAT inhibition. Trichostatin A (TSA), a natural product HDAC inhibitor was used as a positive control. Treated human colon tumour cell lines (HCT116 and HT29) (8000 cells/well seeded) were fixed and permeablised in 96-well plates and primary antibodies with different specificity for histone acetylation added. Following washing, a europium-labelled second antibody (PerkinElmer Life Sciences) was added and time-resolved fluorescence measured. Fluorescent counts were normalised to protein measured in the same well using BCA reagents. Cellular acetylation increased in response to TSA (0.01 – 2.2µM) compared to DMSO treated controls within as little as 15 minutes and results were consistent with those obtained by Western blotting. An acetylated protein antibody (Abcam Ltd.) has been used to determine the extent of cellular acetylation in cells cultured over 5 days. Acetylation peaked 24 and 48hrs following plating out before decreasing again 72hrs - 120hrs later. The relationship between cellular acetylation and proliferation is being studied further using antibodies that recognise different histones and selective sites of acetylation. The ELISA is proving useful as a pharmacodynamic measure of HDAC inhibition *in vitro* and to evaluate and rank HAT inhibitors identified by high-throughput screening. The ELISA is also being used as a cell-based high throughput screen to identify compounds that either increase or decrease acetylation. Supported by Cancer Research UK Programme Grant SP2330/0203 and the Institute of Cancer Research.

**P148**

**IN EXPERIMENTAL METASTASES INSULIN-LIKE GROWTH FACTOR BINDING PROTEIN-4 (IGFBP-4) IS EXPRESSED AT HIGH LEVELS**

Mc Brierty D*, Harmey J, Bouchier-Hayes D

A murine model of metastatic breast cancer has been established. 4T1.2 murine mammary carcinoma cells are a subclone of the spontaneously arising BALB/c 4T1 cell line (a gift from Robin Anderson, University of Melbourne). Lung metastases were established by tail vein injection of 50,000 4T1.2 cells, bone metastases by inter-tibial injection of 10,000 4T1.2 cells. Immunohistochemistry and X-ray imaging were used to confirm that the tumours were bone associated.

Tumour and normal lung and bone tissues were harvested and flash frozen in liquid nitrogen. RNA from the tissues was used to produce radiolabelled cDNA. The labelled cDNA was hybridised against a commercially available gene array containing 588 genes (Clontech), and the arrays were then exposed to X-ray film. Differential gene expression was identified using specialised software (Atlasimage 1.01, Clontech). Housekeeping genes were used on each array to normalise expression patterns between them. A number of genes were putatively identified as being differentially expressed between the bone and lung tumours. These genes were selected for further analysis.

IGFBP-4 was found to be up-regulated in both metastases relative to normal tissues. These results were confirmed through Northern hybridisation and reverse transcription polymerase chain reaction (RT-PCR). IGFBP-4 is involved in the regulation of bone homeostasis. In breast cancer metastasis to bone there is associated bone degredation. Parathyroid hormone, normally released when calcium levels in the blood drop, coincides with an up-regulation of IGFBP-4 in osteoclasts. This IGFBP-4 binds to IGFs, preventing the normal stimulation of osteoblats by IGFs, and therefore decreases osteoblast activity.

**P149**

**THE TUMOUR SUPPRESSOR PTEN AND THE CELL CYCLE INHIBITOR $p27^{KIP1}$ IN PROSTATE CARCINOMA AND PROSTATIC INTRAEPITHELIAL NEOPLASIA (PIN) - AN IMMUNOHISTOCHEMICAL STUDY**

Irina Fenci*, Joachim Woenckhaus, Institut of Pathology, Justus Liebig University, Giessen, Germany

Genetic abnormalities involving the PTEN locus (10q23) and loss of $p27^{KIP1}$ expression are frequently observed in prostate carcinoma, especially in advanced stage disease and metastases. Results of recent investigations on glioblastoma cell lines demonstrated that PTEN determines cell cycle arrest in G1 by activating $p27^{KIP1}$. However, it is currently unknown if a functional relationship exists between PTEN and $p27^{KIP1}$ in the human prostate carcinoma and if this connection can lead to the initiation and/or progression of the disease.

In this study PTEN and $p27^{KIP1}$expression status were immunohistochemically assessed on serial slides in 46 primary prostate carcinomas (23 prostatectomies and 23 transurethral resections), 20 prostatic intraepithelial neoplasias associated to the carcinomas and 15 separate metastases. The results were correlated with the Gleason score (< 7 = low Gleason score, >7 = high Gleason score). We defined a cut-off level for $p27^{KIP1}$ of 40% positive nuclei.

Ten of the 20 PIN lesions (50%) showed loss of at least one marker and $p27^{KIP1}$ was more frequently affected than PTEN (p<0,05). Simultaneous PTEN and $p27^{KIP1}$ deletion was observed in only 1 case (5%). The disease progression from PIN to cancer (assessed by comparing the staining pattern in PIN and cancer lesions of the same case) associated loss of PTEN in 39% of cases and loss of $p27^{KIP1}$ in 15,8% of cases, but no concomitant PTEN and $p27^{KIP1}$ loss.

Nine of 19 carcinomas with low Gleason score (47,3%) showed loss of at least one marker; the same pattern was noticed in 18 of 27 high Gleason scores (66,7%). Seven of 27 high-Gleason scores (25,9%) associated concomitant deletion of both markers, whereas only 2 low-Gleason carcinomas showed this pattern (10,5%). Similar to PIN, the low-Gleason scores correlated with loss of $p27^{KIP1}$ but not with loss of PTEN (p<0,05).

Surprisingly, 10 of the 15 metastases (66,7%) showed positive signals for PTEN , whereas $p27^{KIP1}$ was under 40% (mean 14,2 %) in all but one case.

These data taken together suggest that: 1) loss of $p27^{KIP1}$ occurs earlier and is more frequent than PTEN protein loss in the prostate carcinoma 2) the progression of PIN to invasive carcinoma could be determined by separate loss of PTEN or $p27^{KIP1}$ proteins, but not by a negative regulation of $p27^{KIP1}$ through PTEN protein deletion, 3) the low-differentiated carcinomas and the metastases maintain a surprisingly high PTEN expression, associated with

© 2002 Cancer Research UK

low p27$^{KIP1}$ levels, 4) p27$^{KIP1}$ seems to be down-regulated in prostate carcinoma by other pathways than those involving PTEN.

**P150**

**REGULATION AND INTRACELLULAR LOCATION OF PROTEIN KINASE B/AKT IN HUMAN PROSTATIC CELL LINES**

R Michael Sharrard* and Norman J Maitland, YCR Cancer Research Unit, Dept. of Biology, University of York, York YO10 5YW

Protein Kinase B (PKB, also known as Akt) is a key intracellular signalling enzyme which regulates cell survival, motility, and gene transcription via the apoptotic regulator protein BAD, the Forkhead transcriptional regulators, GSK-3, and caspase 9. PKB is activated by phosphorylation at thr308 and ser473 by phosphatidylinositol-dependent kinases (PDKs). PKB and the PDKs are activated at intracellular membranes by PIP3 generated by phosphatidylinositol 3-kinase (PI3K) in response to soluble growth factors (GFs) and integrin- or cadherin-mediated cell adhesion. PKB is downregulated by PTEN tumour-suppressor protein, which inactivates PIP3.

We studied PKB activation in cell lines derived from human non-tumour prostate epithelium (PNT2, PNT1a) and from prostate tumour metastases (LNCaP, PC3). Phosphorylation of PKB was assayed using antibodies specific to the ser473- and thr308-phosphorylated protein forms in Western blot probing and immunocytochemistry. The non-tumour cells showed strong dependence on soluble GFs for maintenance of PKB activation. EGF (5 ng/ml) caused a rapid (<5 min) peak of activation followed by a gradual (>30 min) decline, while 5 ng/ml IGF-I or IGF-II caused a slower (>30 min) increase to higher final levels of phosphorylation. The tumour cells maintained high levels of PKB activation in the absence of exogenous GFs. Inhibition of PI3K by $10^{-5}$M LY294002 resulted in rapid loss of PKB activation (<30 min) in the non-tumour cells, followed by partial recovery over 2-3 hours. In contrast, the tumour cells showed a slower (3 hours) response to LY294002 which did not reverse. PKB activation in the different cell lines was paralleled by levels of phosphorylation of GSK-3 and by levels of S and G2/M phases and maintenance of cell growth. Confocal microscopy showed that phosphorylated PKB was located at the plasma membrane in the tumour cells, while in the non-tumour cells it was largely associated with particulate or vesicular cytoplasmic structures, where it was resistant to LY294002. Cells transfected with myc-tagged PDK-1 or with the pleckstrin homology domain of PKB fused to green fluorescent protein revealed a similar pattern, indicating that PKB activation and location is PIP3-dependent.

PKB activation in normal prostatic epithelial cells thus appears to be maintained by GF-dependent, LY294002-resistant generation of cytoplasmic vesicle-associated PIP3 by PI3K, while in tumour cells the PIP3 is largely generated at the plasma membrane by PI3K which is independent of exogenous GFs, but extremely sensitive to LY294002. These studies suggest that drugs which inhibit PI3K and its products may be selectively therapeutic in treatment of advanced prostate cancer. Supported by YCR and MRC.

**P151**

**HUMAN SPROUTY2 (A NOVEL INHIBITOR OF FGF RECEPTOR SIGNALLING) IN HUMAN PROSTATE CANCER**

Douglas DA*, Mehta PB, Neal DE, Leung HY
Prostate Research Group, Department of Surgical Sciences, Medical School, University of Newcastle upon Tyne. NE2 4HH

**INTRODUCTION:**

The Fibroblast Growth Factors (FGFs) and their receptors (FGFRs) are important in prostate cancer pathogenesis. Over-expression of multiple members of the FGF family (FGF1, FGF2, FGF7 and FGF8), occur in prostate cancer. FGFs activate FGF Receptors. FGFR1 and FGFR2 are over-expressed in prostate cancer.

Sprouty protein was first described in *Drosophila* to regulate airway development. It functions in a competitive fashion to block FGF mediated signalling. It has similar activities in vertebrates. The expression of Sprouty is induced by activation of FGFR and therefore serves as a physiologic negative feedback to control the function of activated receptors. Over-expression of Sprouty blocks FGF mediated signalling enough to result in an altered phenotype. We aimed to examine expression levels of hSprouty2 in human prostate cancer.

**MATERIALS AND METHODS:**

RNA was extracted from 62 BPH and malignant prostate chips. Expression of hSprouty2 was examined within prostate samples using Reverse Transcription (RT)-PCR and Western Blot analysis. Sprouty mRNA expression was quantitated by Real Time RT-PCR, which is currently the most sensitive method for mRNA quantitation.

**RESULTS:**

Expression of hSprouty2 was compared between benign prostate hypertrophy (BPH), low grade (Gleason 2-4), moderate grade (Gleason 5-7) and high grade (Gleason 8-10) prostate carcinoma. Levels in BPH and low grade carcinoma were similar, and although moderate grade carcinoma showed a downward trend in Sprouty expression, this was not significant. When compared to the BPH group, hSprouty2 mRNA expression was significantly reduced in high grade tumours ($p<0.005$).

**CONCLUSIONS:**

Expression of hSprouty2 (a physiologic inhibitor of FGFR) is significantly down-regulated in high grade prostate cancer. The functional significance of such hSprouty2 down-regulation warrants further investigation.

**P152**

**METHYLATION OF GSTP-1, CD44, E-CADHERIN AND RASSF1A IN NORMAL AND MALIGNANT PROSTATE TISSUE**

K Patel*[1], K MacLennan[1], PJ Selby[1], S Burchill[1]
[1]Division of Cancer Medicine Research Cancer Research UK Clinical Centre, St. James's University Hospital, Leeds, U.K. e-mail: medkp@cancermed.leeds.ac.uk

Sensitive methods for the detection of prostate cancer may be useful in defining prognosis and in directing future therapeutic strategies. RT-PCR for PSA mRNA in the peripheral blood of cancer patients has been studied by our group and others and found it to be sensitive but lacking in absolute specificity. Identification of tumour specific molecular abnormalities should circumvent this problem, however consistent tumour specific abnormalities have not been identified in prostate cancer. Abnormal methylation of 5' CpG islands within promoter regions of tumour suppressor genes have been identified in prostate cancer (GSTP1, CD44 and e-cadherin) and proposed as a mechanism of inactivation of these genes. These tumour specific abnormalities within GSTP1, CD44 and e-cadherin were studied further in prostate tumours as well as RASSF1A a putative tumour suppressor gene in bladder and nasopharyngeal cancers. DNA from eleven laser-microdissected prostate tumour needle biopsies and 5 non-malignant prostate biopsies were modified with bisulphite and methylation examined using a sensitive nested methylation -specific PCR.

GSTP1 methylation was detected in all tumours, CD44 methylation in 5/11 tumours, e-cadherin methylation in 1/11 tumours and RASSF1A methylation in 10/11 tumours.

Non-malignant prostate biopsies from men with elevated serum PSA levels also showed methylation patterns for all 4 genes.

The methylation status of GSTP1 and CD44 concurs with other studies in prostate cancer, however e-cadherin methylation was rarely seen. RASSF1A was also methylated in prostate cancer (novel observation).

Interestingly, methylation of these genes was also found in age-matched non-malignant prostate tissue. We hypothesise that the methylation patterns seen in normal tissues may be an age-related change or be part of a spectrum of 'background' changes seen prior to the malignant phenotype. Tumour specificity for methylation of the 4 genes studied was not demonstrated.

© 2002 Cancer Research UK

**P153**

**INVESTIGATION OF THE INVASIVE CAPACITY OF BASAL CELL CARCINOMA USING AN INVASION ASSAY MODEL**

P. Lim*[1], GD Wilson[2], R Sanders[1]
[1]RAFT Institute of Plastic Surgery & Gray Cancer Institute
[2]Mount Vernon Hospital, Northwood, Middlesex

Basal cell carcinoma (BCC) is the commonest cancer in humans and presents major problems in terms of surgery for the patient and workload for the NHS with over 40000 cases of BCC each year in the UK.
Despite this there has been relatively little research into BCC compared to other skin cancers. We have previously shown that in aggressive BCC there is an increased expression of matrix mealloproteinase-2 (MMP-2) and reduced expression of its inhibitor TIMP-2. MMPs have been implicated in the invasion and metastasis process in a number of different tumours. We are examining if the invasive properties of BCC and investigating if these can be modified with inhibitors of MMPs.
Invasion studies were performed using the cell line of BCC, KMC-1/BCC and primary cell lines isolated from surgical specimens. Suspensions of the BCC cells were placed in the upper compartment of the invasion chamber and these were attracted to conditioned media in the lower chamber. Invasive cells that migrated through matrigel-coated filters were stained and counted.
It has been possible to use this experimental model to examine the effect of a number of substances on BCCs including TIMP-2 and synthetic MMP inhibitors such as ilomastat. Provisional results suggest that the invasive capacity of BCC can be reduced by MMP inhibitors.

**P154**

**CHANGES IN THE APOPTOTIC PHENOTYPE OF PROSTATIC EPITHELIAL CELLS DURING THEIR DIFFERENTIATION FROM BASAL TO LUMINAL SECRETORY**

Declan G Murphy*, John M Fitzpatrick, R. William G. Watson
Department of Surgery, Mater Misericordiae Hospital, Conway Institute of Biomolecular and Biomedical Research, University College Dublin, Ireland.

Basal prostate epithelial cells differentiate into an endstage luminal cell which is dependent on the presence of androgen. Why these cells become androgen dependent and susceptible to cell death via apoptosis remains unknown. We hypothesise that alterations in the apoptotic phenotype of the luminal prostate epithelial cells ultimately leads to its dependence on androgen and susceptibility to apoptosis.
The NRP-152 cell line is a benign basal cell line derived from the dorsal-lateral prostate of the rat. Under mitogen-deficient conditions, NRP-152 cells were differentiated from a basal to a luminal prostate epithelial cell. Using Western Blotting immunofluorescence, this change in phenotype was confirmed by the progessive loss of cytokeratin 14 expression and the increased expression of cytokeratin 18, over an 8 day time-course. The expression of the apoptotic proteins Bcl-2, MCL-1, cIAP-1, cIAP-2 and caspase 3 were then characterised. Western blotting demonstrated that the anti-apoptotic proteins Bcl-2, MCL-1, cIAP-1 and cIAP-2 all decreased in expression in a time dependent manner. However, the expression of the pro-apoptotic protein, caspase-3, was unchanged.
These findings suggest that prostatic luminal cells become more susceptible to apoptosis during differentiation as a result of decreased expression of anti-apoptotic proteins. Further characterisation of these apoptotic changes will help define the potential areas in the differentiation process where carcinogenesis and hormone-independence may occur.

**P155**

**CELL CYCLE CHECKPOINT RESPONSE AND MICRONUCLEUS FORMATION INDUCED BY UVA RADIATION IN CELLS FROM INDIVIDUALS HARBOURING GERMLINE MUTATIONS IN BRCA1**

S.E. Tobi[1]*, J. Shorrocks[1], R. Eeles[2], J.H.Peacock[2] and T.J.McMillan[1]
[1]Dept. of Biological Sciences, Lancaster University, Lancaster LA1 4YQ, [2] Institute of Cancer Research, Sutton, Surrey, SM2 5NG

Individuals carrying germline mutant alleles of the BRCA1 gene have an increased risk of developing breast and ovarian cancer. A number of observations point to the participation of the BRCA1 protein in the cellular response to DNA damage and, more specifically, there is evidence for its involvement in regulating cell cycle checkpoints after ionising radiation. Using primary human diploid fibroblasts from individuals either wild-type for BRCA1 or containing a mutant allele (heterozygous), we examined the cell cycle response following exposure of exponentially–growing cells to the oxidative stress induced by UVA radiation (320-400nm). Although cells from all individuals exhibited a G1 arrest after UVA ($100kJ/m^2$ ), the response was of a shorter duration in the BRCA1 (+/-) heterozygotes. Thus, 14 hours after irradiation, the proportion of cells in S-phase in three wild-type fibroblast strains (expressed as percentages (mean ± range) of corresponding unirradiated values) was 20.33 ± 5.36, 16.85 ± 2.25 and 7.85 ± 2.41, as compared to values of 33.78 ± 1.52 and 37.53 ± 1.52 for two BRCA1 (+/-) strains. Since a defective G1/S checkpoint in BRCA1 heterozygotes could lead to a greater proportion of S-phase cells with unrepaired DNA damage (strand breaks) and a resultant increase in chromosomal instability, the frequency of micronuclei induced by UVA was examined. Three normal (+/+) and three mutant (+/-) strains (two of which were used in the cell cycle experiments) produced mean micronuclei frequencies of 0.080 ± 0.013 and 0.094 ± 0.031/binucleate cell respectively (not statistically significant), 48h after UVA exposure . Our data suggest a defective G1/S checkpoint in cells from BRCA1 heterozygotes in response to UVA radiation although this is not reflected in genomic instability as measured by micronuclei induction.

**P156**

**INTRACELLULAR SIGNALING IN THE NFκB ACTIVATION PATHWAY FOLLOWING OXIDATIVE STRESS MEASURED BY FLUORESCENCE LIFETIME IMAGING OF FLUORESCENT PROTEINS**

Sarah A. Roberts*[1], Simon M. Ameer-Beg[2], Borivoj Vojnovic[2] and Peter Wardman[1], Free Radicals in Cancer[1] and Advanced Technology[2] Groups, Gray Cancer Institute, PO Box 100, Mount Vernon Hospital, Northwood, Middlesex, HA6 2JR, UK

Fluorescence resonance energy transfer (FRET) between spectrally different chromophores has allowed the study of protein-protein interactions to be followed within the cell. In this study, spatially resolved FRET was measured by multiphoton fluorescence lifetime imaging microscopy, which provides a method for following changes in the lifetimes of fluorescently tagged proteins, indicating a change in protein environment inside both live and fixed cells.
NFκB is a transcription factor that is activated by a wide variety of stimuli including ionizing radiation and many anticancer agents. Its activation has been shown to be cytoprotective and may reduce the efficacy of many anticancer drugs. Oxidative stress has also been shown to activate NFκB, but only in certain cell lines. However, in all cell lines examined NFκB activation was inhibited by the addition of antioxidants. In order to determine whether reactive oxygen species are involved in NFκB activation and where in the NFκB pathway they exert their effect, we have used green fluorescent protein (GFP) tagged fusion proteins of NFκB and other members of the signalling pathway to examine lifetime changes following a variety of stimuli inducing oxidative stress. Measurements were performed by analysis of fluorescence decay kinetics acquired using single photon counting following two-photon excitation (*ca* 150 fs 890 nm pulses), on a pixel-by-pixel basis.
We initially examined the interaction between IκBα (inhibitory subunit of NFκB) and its activating kinase IKK in MCF-7 cells transiently transfected with both GFP fused IκBα and FLAG-tagged IKK. Following treatment, the cells were fixed and the IKK protein was labelled with AlexFluor 532. By stimulating of the cells with activators of NFκB, a reduction in the average lifetime of GFP was observed indicating the proteins change in the environment. This reduction was only observed in the cytoplasm of the cell (not in the nucleus) confirming the cytoplasmic localization of the IκBα/IKK interactions. When lifetime data were fitted with biphasic kinetics quantitative measurements of FRET efficiency were obtained which allowed measurements of the distance between the two proteins during interaction. Oxidative stress (100 μM $H_2O_2$) was able to stimulate members of the NFκB activation pathway in this MCF-7 cell line as measured by FRET and confirmed by Western Blot analysis of NFκB and IκBα proteins. In conclusion, the results show that analysis of the lifetime images was successful in reporting interactions between the proteins IκBα and IKK.
*This work is supported by the Cancer Research UK.*

© 2002 Cancer Research UK

**P157**

**THE EFFECT OF CONJUGATED LINOLEIC ACID (CLA) AND ITS ISOMERS ON THE PROLIFERATION, MIGRATION AND INVASION OF A METASTATIC MURINE MAMMARY CELL LINE**

Áine O'Connor*, Susan McDonnell[1], Rosaleen Devery[1], Catherine Stanton[2].
School of Biotechnology, [1]National Institute for Cellular Biotechnology, Dublin City University, Dublin 9, Ireland and [2]Teagasc Dairy Products Research Centre, Moorepark, Fermoy, Co. Cork, Ireland.

Conjugated Linoleic Acid (CLA), a polyunsaturated fatty acid, refers to a group of dienoic derivatives of linoleic acid that can be found in natural food sources, such as milk fat and the meat of ruminant animals. CLA has been shown to have anti-carcinogenic activity in many *in vitro* and *in vivo* studies. Previous studies have focused on the effects of dietary CLA on the prevention of tumour appearance, yet relatively little is known about the actual mechanism of CLA's anti-cancer activity.
The most lethal aspect of cancer is the ability of tumour cells to metastasise and form secondary tumours. The matrix metalloproteinases (MMPs), a multi-gene family of enzymes, degrade components of the extracellular matrix (ECM). The MMPs have been implicated as major role players in tumour invasion and metastasis. The aim of this study was to examine the effect of CLA on the growth, MMP-9 expression and invasive activity of a murine mammary tumour cell line, 4T1, which is known to be highly metastatic *in vivo*.
Cells were treated with CLA, which contains a mixture of various isomers, and with the purified predominant isomers present in CLA, 9*c*,11*t*(18:2) and 10*t*,12*c*(18:2). Cytotoxicity was examined using a Promega Proliferation assay, with varying CLA concentrations and time points chosen. Sub-lethal as well as lethal doses were determined. Interestingly, it appeared that the 10*t*,12*c*(18:2) isomer had the most lethal effect, killing the cells with an $IC_{50}$ of 13μg/mL when incubated for 2 days. In contrast, the CLA mix and the 9*c*,11*t*(18:2) isomer had an $IC_{50}$ of 40 and 20 μg/mL, respectively, when incubated for 4 days.
Treatment of the cells with a sub-lethal dose of CLA and its isomers also resulted in a reduction in the invasive activity of the cell line. This was assayed by coating Matrigel (Sigma), which acts as ECM, onto 8μm pore-size membranes through which cells invade *in vitro*. Whilst, the CLA mix reduced invasion compared to control (ethanol) treated cells, the isomers again appeared more potent than the CLA mix. Using substrate zymography, which measures MMP activity, the amount of MMP-9 expressed by the 4T1 cells was also reduced by treatment with CLA and its isomers.
Both the invasion assays and the MMP analyses indicate strongly that CLA may be used as an anti-metastatic agent *in vitro*. *In vivo* work using this fatty acid and this cell line, for the induction of lung nodules, will be the next step in elucidating the importance of CLA in anti-cancer treatments.

**P158**

**THE ROLE OF CD44 AND EZRIN DURING PROSTATE CANCER-ENDOTHELIUM INTERACTION.**

Gregory M. Harrison*[1], W. G. Jiang[1], M D Mason[2]. [1]*Departments of* Surgery and [2]Clinical Oncology, University of Wales College of Medicine Cardiff. UK.

CD44 is a multifunctional cell surface adhesion molecule that has been implicated in tumour cell invasion and metastasis. Many cancer cell types as well as their metastases expresses high levels of CD44. It is known that ezrin, a member of the ERM family of proteins, can bind to CD44 and thus raises the possibility that it is involved in cell migration and metastasis. Therefore we examined the expression and distribution of CD44, its co-localisation and translocation with ezrin in prostate cancer cell lines as they interact with endothelial cells.
RT-PCR revealed multiple isoforms of CD44 were expressed in DU-145 and PC-3 and DNA Sequencing identified both the standard and different variant isoforms in both cells. CD44 and ezrin were found to associate with each other in prostate cancer cell lines as seen by immunoprecipitation and Western blotting. In addition, incubation of cells with Hepatocyte growth factor/scatter factor (HGF/SF), a cytokine know to enhance cancer invasion and migration, resulted in the up regulation of CD44 expression in both PC-3 and DU-145 cells as seen by Western blotting. Dual-immunofluorescence and laser scanning confocal microscopy revealed the co-localisation of CD44 and ezrin but also the co-translocation of these proteins in response to HGF/SF exposure. Using a tumour-endothelial adhesion assay, more HGF/SF-activated PC-3 cells were found adhered to endothelial cells than untreated PC-3 cells and adhesion was further increased by pre-activated endothelium with HGF/SF. Results from an *in vitro* invasion assay confirm a significant increase in the invasive potential of HGF-treated cells. The increased adhesion and invasiveness were reduced or abolished by the inclusion of anti-CD44 antibodies or the addition of an excess hyaluronic acid (HA).
In conclusion, the change in distribution of CD44-Ezrin complex and up-regulation of CD44 suggest CD44 plays a role in the initial capture and subsequent invasion of endothelium by prostate tumour cells.

**P159**

**A CELL MODEL SYSTEM TO STUDY THE MOLECULAR BASIS OF EXT MUTATIONS USING *IN VITRO* CHONDROGENESIS**

Helen C.M. Leung*, Danny Chan, Kathryn S.E. Cheah, and Daisy K.Y. Shum
Department of Biochemistry, The University of Hong Kong, Hong Kong, China

Mutations in *Ext1* and *Ext2* are associated with Hereditary Multiple Exostoses (HME), which is characterized by benign cartilage-capped outgrowths (exostoses) from ends of long bones. The Ext1 and Ext2 proteins possess a copolymerase activity that participates in heparan sulphate (HS) chain extension on the linker tetrasaccharide to the core protein of proteoglycans (PGs). Cell surface PGs can interact with diffusible growth factors (FGFs) and signaling molecules (Ihh) expressed in the growth plate cartilage and thus play a part in regulating bone development. A hypothesis is that mutations in the *Ext1* and *Ext2* genes alters HS synthesis, in terms of the length of HS chains and sulphation pattern, leading to defective chondrogenesis as a prelude to the formation of exostoses.
Previous studies on *Ext1* and *Ext2* mutations have demonstrated a number of mutations act in a dominant-negative manner leading to defective HS chain synthesis. However, these studies were carried out in non-chondrogenic cell line. Our objective is to establish tools and a cell based model system required to study the molecular and cellular changes during chondrogenesis. In this system, we will exploit the ability of a mouse chondrogenic progenitor cell line, ATDC5 that undergoes *in vitro* chondrogenesis in culture. We have initiated stable transfections with expression plasmids for wild type *ext1* and *ext2* cDNA, and cDNA plasmids with dominant-negative mutations in *ext1* (R340C) and *ext2* (D227N) genes. This will allow the opportunity to monitor the effect of the mutations on proteoglycan synthesis. In particular, extension of haparan sulphate chain and sulphation pattern. More importantly, we can monitor and compare the progression of *in vitro* chondrogenesis and to address the responsiveness of transfected cells to Indian Hedgehog (Ihh) signaling. Experiments are also in progress to establish efficient protocol to monitor haparan sulphate synthesis using a newly developed fluorescent-assisted carbohydrate electrophoresis.

**P160**

**INTERACTIONS BETWEEN CD44, BETA-1 INTEGRIN AND THE MATRIX METALLOPROTEINASES IN REGULATING COLORECTAL CANCER CELL ADHESION AND INVASION**

D. Murray*, S. McDonnell
School of Biotechnology, National Institute for Cellular Biotechnology, Dublin City University, Dublin 9, Republic of Ireland.

The complex process of tumour cell invasion involves the attachment of cells to the extracellular matrix (ECM), degradation of the ECM and subsequent migration and detachment of cells from the ECM. Cell adhesion molecules like CD44 and integrins are key players in such cell-cell, as well as cell-matrix, adhesion interactions. Beta–1 integrin mediated signal transduction is thought to up-regulate matrix metalloproteinase (MMP) expression via the mitogen activated protein kinase (MAPK) pathway. Collectively, members of the MMP family can degrade the ECM completely. Such extracellular proteolytic activities have given the MMPs well established roles in tumour cell migration and invasion. Appreciating the importance of both attachment

© 2002 Cancer Research UK

to and degradation of the ECM in tumour cell invasion, it is no longer appropriate to consider CD44, integrins and MMPs as acting in isolation. This research aims to elucidate the interactions of these molecules in regulating colorectal tumour metastasis.
The expression of CD44 and beta-1 integrin was examined in the primary human colon carcinoma cell line SW480, its lymph node metastatic derivative SW620 and SW480(Mut-6) which have been transfected with the gene for Matrilysin (MMP-7). The SW480(Mut-6) has been shown to be more invasive than the parental SW480. Western blot analysis showed that the SW480, SW620 and SW480(Mut-6) expressed an 80kDa variant of CD44 with increased expression in the SW620. The expression of a 120kDa variant of CD44 was also observed in SW620 but not in SW480 or SW480(Mut-6). The SW620 cell line also expressed higher levels of Beta-1 integrin than the other cell lines. The adhesion of these cell lines on plastic, fibronectin and hyaluronic acid was also examined. Results showed that there was a significant difference in cell attachment between these cell lines. The SW620 and SW480(Mut-6) were more adherent to plastic than the parental SW480. The SW480(Mut-6) were also shown to be more adherent to fibronectin and hyaluronic acid than SW480. In contrast, the SW620 appeared to be less adherent to hyaluronic acid than SW480. These results demonstrate that MMP-7, CD44 and beta-1 integrin interact with each other to regulate colorectal tumour cell attachment and migration. This work will help elucidate the functional relationships of these molecules in controlling invasion and metastasis in colorectal cancer.

**P161**
**THE BISPHOSPHONATE ZOLEDRONIC ACID UPREGULATES APOPTOTIC SIGNALS AND IMPAIRS THE ADHESION OF BREAST CANCER CELLS TO MINERALISED MATRICES PRIOR TO THE INDUCTION OF APOPTOSIS**
Lisa M Pickering*, Siddhika G Senaratne, Janine L Mansi, Kay W Colston
St. George's Hospital Medical School, Cranmer Terrace, London. UK. SW17 0RE.

**AIMS.** Bisphosphonates are of proven efficacy in limiting skeletal related morbidity in patients with advanced breast cancer. The mechanisms by which these compounds exert their clinically important effects remain inadequately understood. We have previously demonstrated that bisphosphonates directly induce apoptosis in the breast cancer cell lines MCF-7 and MDA-MB-231[1]. We aimed to further elucidate the signalling pathways of these effects. We also aimed to investigate the relationship between bisphosphonate induced breast cancer cell apoptosis and effects on adhesion to bone-like dentine matrices.
**PROCEDURES.** Breast cancer cells were incubated in medium supplemented with zoledronic acid or vehicle. Apoptosis was assessed morphologically and by colourimetric assays of cell proliferation and cell viability. Immunoblotting was performed using a standard assay comprising gel electrophoresis, transfer to a nitro-cellulose membrane, protein detection with primary and secondary antibodies and protein visualisation against colour standards by chemiluminescence. Adhesion assays were performed by seeding treated breast cancer cells onto mineralised matrix for 1 – 24 hours. Adherent cells were fixed, stained and counted.
**MAJOR FINDINGS.** Zoledronic acid induced apoptosis in the breast cancer cell lines MDA-MB-231, MCF-7, SKBR3 and HS578T. Treatment of MDA-MB-231 and MCF-7 breast cancer cells with 100 μM zoledronic acid resulted in downregulation of the anti-apoptotic protein bcl-2 and upregulation of the pro-apoptotic protein bax. The importance of bcl-2 as a survival signal was confirmed by the demonstration that forced bcl-2 expression in MDA-MB-231 cells abrogated the effects of zoledronic acid. Pro-caspase 3 cleavage was demonstrated by a FACS based assay in MDA-MB-231 cells but not in MCF-7 cells which constitutively lack caspase 3. However, MCF-7 cells underwent a time-related cleavage of caspase 7 in response to treatment with zoledronic acid. Finally we provide evidence to suggest that zoledronic acid can impair the normally observed adhesion of breast cancer cells to mineralised matrix in a dose and time dependent manner and moreover that this occurs at an earlier time point than that required to observe its apoptotic effects.
**SIGNIFICANCE OF RESEARCH.** Our findings suggest that clinical responses to bisphosphonates in patients with breast skeletal metastasis are related to induction of apoptosis. This may occur through impaired cell-matrix interactions resulting in anoikis.
**CONCLUSIONS.** We suggest that zoledronic acid-induced impairment of breast cancer cell adhesion occurs prior to induction of apoptosis and anoikis may play a role in bisphosphonate induced cell death. The precise effector signals of this pathway remain to be determined.
1. Senaratne SG, Pirianov G, Mansi JL, Arnett TR, Colston KW. 2000. Br J Cancer;82:1459

**P162**
**HES6 REGULATES MYOGENIC DIFFERENTIATION**
Cossins, J[1], Vernon, A E[2] , Zhang, Y[1], Philpott A[2] and Jones P H[2] *,
1 Cancer Research UK, Weatherall Institute of Molecular Medicine, John Radcliffe Hospital, Oxford, UK. 2 Cancer Research UK Department of Oncology, University of Cambridge, Hutchison/MRC Research Centre, Addenbrooke's Hospital, Cambridge, UK

In rhabdomyosarcoma, tumour cells fail to undergo the cell cycle withdrawal and terminal differentiation that occur in normal muscle differentiation. Candidate regulators of proliferation and differentiation in normal and malignant myogenesis are the Hes proteins, basic helix-loop-helix transcription factors homologous to Enhancer of Split (EoS) proteins that regulate differentiation in Drosophila.
We identified Hes6 as a member of the Hes family expressed in normal muscle and rhabdomyosarcoma by database searches. Hes6 is known to promote neural differentiation and to bind to Hes1, a related protein that has been implicated in cell cycle regulation, affecting Hes1 function. Here, we show that Hes6 is expressed in the murine embryonic myotome and is induced on C2C12 myoblast differentiation in vitro. Hes6 binds DNA containing the Enhancer of Split E box (ESE) motif, the preferred binding site of Drosophila EoS proteins, and represses transcription of an ESE box reporter. When overexpressed in C2C12 cells, Hes6 impairs normal differentiation, causing a decrease in the induction of the cyclin dependent kinase inhibitor, $p21^{Cip1}$, and an increase in the number of cells that can be recruited back into the cell cycle after differentiation in culture.
Hes6 is well conserved in Xenopus, so we used this system to study the effect of Hes6 on myogenesis in vivo. In situ hybridisation in embryonic Xenopus reveals that Hes6 is co-expressed with the early muscle marker, MyoD. Microinjection of Hes6 RNA in Xenopus embryos results in an expansion of the myotome, but suppression of terminal muscle differentiation and disruption of somite formation at the tailbud stage, paralleling the phenotype seen in vitro. Analysis of Hes6 mutants indicates that whilst the DNA binding activity of Hes6 is not essential for its myogenic phenotype, protein-protein interactions mediated by the C terminus of the protein are required.
Thus we demonstrate a novel role for Hes6 in multiple stages of muscle formation, and that down regulation of Hes6 expression is required for terminal differentiation of muscle. We plan to investigate if Hes6 is highly expressed in rhabdomyosarcoma and identify Hes6 interacting proteins.

**P163**
**METHIONINE DEPENDENCY OF TUMOURS: A BIOCHEMICAL STRATEGY FOR SYNCHRONISING CELLS IN SPECIFIC PHASES OF THE CELL CYCLE.**
V Pavillard*, DJ Swaine, RM Phillips, A Nicolaou, & JA Double
Cancer Research Unit and School of Pharmacy, University of Bradford, Bradford BD7 1DP, UK.

A well established feature of tumour cell proliferation (Hoffman BBA 1984, 738: 49-87) is their dependence upon the essential amino acid methionine (MET) whereas normal cells are MET independent. Tumour cells cultured in medium which is deprived of MET but supplemented with its precursor, homocysteine (HCY) show a marked decrease in cell proliferation in conjunction with a distinct accumulation of cells in the S/G2 phase. Release of the cell cycle blockade by addition of MET (following a period of MET starvation) has not been previously studied. It offers the potential of synchronising cells in a specific phase of the cell cycle thereby rendering cells more susceptible to cell cycle phase specific agents and producing therapeutic gain. The cell lines used in this study were rat hepatocarcinoma lines HTC (MET dependent), Phi-1 (partially MET independent) and the human skin fibroblast cell line, Hs-27 (MET independent). Under standard

© 2002 Cancer Research UK

